# HB E/β°Thalassemia can present with normal phenotype

**DOI:** 10.4103/0973-6247.67023

**Published:** 2010-07

**Authors:** Ranabir Pal, Subrata Bagchi, Sumit Kar, Shrayan Pal

**Affiliations:** Department of Community Medicine, Sikkim-Manipal Institute of Medical Sciences (SMIMS) and Central Referral Hospital (CRH), 5^th^ Mile, Tadong, Gangtok, Sikkim. 737 102, India; 1 Department of Community Medicine, NRS Medical College, Kolkata, India; 2 MBBS Student, Sikkim-Manipal Institute of Medical Sciences (SMIMS), India

Sir,

Hb E is the second most common hemoglobin variant in the world with a high frequency in SE Asia reaching a frequency from 15% to 50% in Thailand, Burma, Srilanka, and Vietnam as the most common hemoglobin variant. Hemoglobin E/β°Thalassemia, however, resembles homozygous β°Thalassemia both clinically and hematologically. Heterozygous HbE diseases are asymptomatic with mild hypochromia that is prevalent in Bengal, Assam, and in East India. Homozygous suffer from mild hemolytic anemia and have modest splenomegally with target cell 25-50%. HbE diseases entity often goes unnoticed as most of the cases have no noticeable clinical findings as reviewed extensively by Wasi in Thailand.[1–5]

A 6 years old girl presented with progressively increasing generalized weakness, difficulty in walking with breathlessness, withdrawn at home, poor appetite since her third birthday. Family history or other histories revealed no significant related events of regular intake of blood.

The girl was moderately pale, mild icteric with mesomorphic features with an apparently normal look for her age and sex with mild hepatosplenomegally. No dysmorphic feature (mega hepatosplenomegally or bronzy discoloration of skin or typical facial changes) suggestive of chronic or congenital hematological diseases was present to bring the child under multiple differential diagnoses. Pulse oxymetry showed 91% oxygen saturation. Hemoglobin level was 4 g% with few reticulocytes, numerous hypochromic nucleated red cells, and microcytes with almost no normal appearing red cells on smear, occasional spherocytes present, more than 50% red cells being the target cells. MCV was 50-66 fL and serum bilirubin 1.84 mg%, unconjugated 1.4, conjugated 0.4. The hemoglobin agar gel electrophoresis at pH 8.6 showed Hb E in the same position as Hb C and Hb A_2_. At pH 6.3, Hb E ran with Hb A; HbA 0.2 g (5.8%), HbF 1.8 g (44.1%), HbE 2.0 g (50.1%). The caregivers were counseled with the ‘live with a disease’ philosophy and the diagnosis as well as prognosis of this disease was carefully explained in the line of management of β°Thalassemia.

It is very important to generate awareness among our fraternity regarding subtle presentation and importance of early detection of E/β°Thalassemia and that timely intervention can help in better prognosis. Professionals related to community pediatrics, clinical epidemiology, and community genetics, functioning in unison, has a dominant role to play in preventing the fatal outcome of this genetic disorder.
